# Assessing Emotional Expressions During a Cycling-Based Initiative for Older Care Home Residents Using Video-Based Recordings

**DOI:** 10.1177/23337214221099689

**Published:** 2022-08-09

**Authors:** Ryan Gray, Shana Faraghat, Alan J. Gow

**Affiliations:** 1 Centre for Applied Behavioural Sciences, Department of Psychology, School of Social Sciences, 3120 Heriot-Watt University, Edinburgh, UK

**Keywords:** emotional expressions, video-based observation, care home, supported living

## Abstract

**Objective:** Through Cycling Without Age, trained volunteers use specially designed trishaws to provide rides for older adults living in care homes and other supported living environments. Qualitative and quantitative research suggests benefits in terms of improvements in mood and wellbeing. Those studies have predominantly been interviews with participants reflecting on previous rides, or as pre-/post-assessments. The current study assessed emotional experiences using video recordings acquired during participants’ rides. **Methods:** Twelve older adults (50% female; 67-92 years old (M = 81.8, SD = 7.4)) living in care homes or supported living environments were recruited. During a Cycling Without Age ride, participants were filmed using an action camera mounted on the trishaw. Recordings were rated using the Facial Expression Coding System by two researchers to assess the frequency, duration and intensity of positive and negative emotions. **Results:** On average, 23.7 positive emotional expressions were observed per ride, significantly higher than negative emotions (0.4). As well as more frequent, positive emotions were observed over a longer duration in total (139.5 seconds vs. 1.3) and rated as more intense (1.9 out of 5 vs. 0.3). **Conclusion:** The study supported the value of directly assessing emotional responses during this cycling-based initiative, including minimising the input required from participants. The predominantly positive emotional expressions observed were consistent with both qualitative and quantitative assessments of Cycling Without Age, and suggests a potential pathway by which those benefits manifest. Future studies might adopt a triangulated approach, using in-activity monitoring, quantitative assessments and participant reflections.

## Introduction

**Figure 1.**
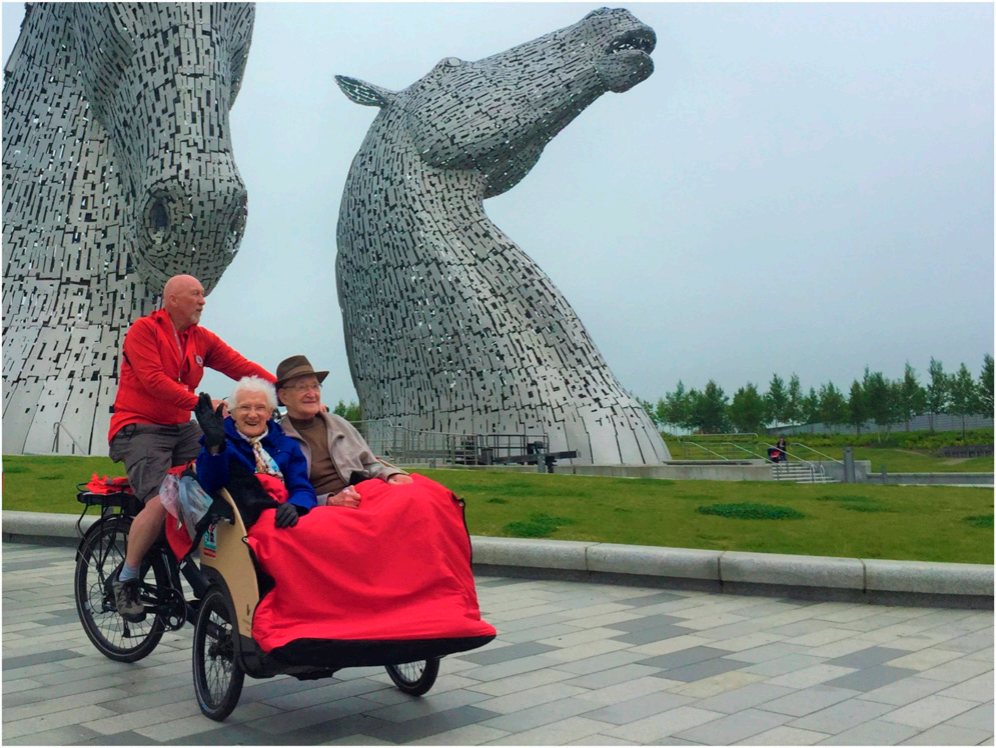
Cycling Without Age trishaws are piloted by a trained volunteer (referred to as a pilot and seated at the rear) with up to two passengers seated up front. Photo credit: A. Gow. Image cannot be reproduced without permission.

Cycling Without Age (CWA) is a charity initiative in which trained volunteers (known as pilots) use specially designed trishaws to take people on rides exploring their local area (Figure 1). Those who benefit are primarily older adults living in care homes and supported living environments, many of whom have limited mobility and/or cognitive impairments. Since its development in Denmark, CWA has expanded globally. Although research exploring the potential benefits of the initiative remains limited, improvements in the mental wellbeing and quality of life of care home residents have been reported (Gow et al., 2019; Gray & Gow, 2020; McNiel & Westphal, 2020; Salas, 2018). Qualitative findings have highlighted positive perceptions of the initiative in terms of access to fresh air, socialising, relaxing and the relationship formed with CWA volunteers (Gow et al., 2019; McNiel & Westphal, 2020).

Though the available studies are positive, all adopt methodologies that evaluate and measure the impact of CWA within the context of interview-based or pre-/post-test designs. These designs cannot examine the impact of the activity as it happens and limits participation to people capable of answering interview or survey questions. A more inclusive and wide-ranging approach that allows for the measurement of participants’ experiences in real-time would therefore provide a complementary addition to CWA evaluations.

Video-based observation may be one such method to achieve this, but is often underutilised as a data collection tool due to confidentiality and privacy issues (Asan & Montague, 2014). Within healthcare research, consultations between patients and doctors are recorded and their interactions are subsequently analysed (Asan & Montague, 2014). These recordings are beneficial as researchers are able to capture interactions between the participant and their environment, coding both verbal and nonverbal cues (Wang & Lien, 2013). Importantly, it also allows data to be collected from complex and dynamic environments independent of the researcher’s influence.

The current study explored the feasibility of capturing emotional experiences during a CWA ride. An action camera was attached to a CWA trishaw, and independent coders rated the recordings for the number, intensity and valence of participants’ facial expressions throughout their ride. In doing this, the design provided the opportunity to gather a more authentic and detailed understanding of the activity, complementary to other methods. Given the benefits for mood and wellbeing highlighted in the CWA literature, we expected participants to express more frequent and intense positive facial expressions than negative.

## Methods

### Participants

Fourteen older adults living in care homes/supported living environments in Scotland were recruited. However, two recordings were not usable, resulting in a sample of twelve participants aged 67-92 years old (M = 81.8, SD = 7.4; 50% female). As an exploratory study, detailed inclusion/exclusion criteria were not implemented, other than participants being able to provide informed consent and that there were no contraindications to their participation in a CWA ride. Participants’ experience with CWA varied from having never taken part before to over 50 rides (M = 8, SD = 13.6). Three participants had a diagnosis of dementia and three had limited mobility that required the use of a walker; the remaining six participants were predominantly independent and did not require assistance with mobility. 

### Procedure

Participants provided written consent for their CWA ride to be recorded and coded, and reported their age, gender and the number of rides they had previously been on. The researcher attached an action camera to a trishaw panel using a flexi-clamp. As two participants were recorded on some rides, the camera was adjusted to capture both passengers’ facial expressions as clearly as possible. Given the camera was located on one side of the trishaw, the angle was often preferable for capturing the expressions of the closest participant.

Participants were taken on a trishaw ride by a trained CWA pilot. Rides varied in length depending on the wishes of the passengers, the weather and available routes, though usually lasted between 30-40 minutes. The video footage was analysed by two independent coders using the Facial Expression Coding System (FACES) (Kring & Sloan, 2007).

### Materials

The Facial Expression Coding System (FACES) (Kring & Sloan, 2007) is a validated measure of facial expressiveness used with diverse populations including children, healthy older adults (Borrego et al., 2021) and people with dementia (Oliver et al., 2020).

For each recording, coders noted the time at which a participant’s face changed from a neutral position to an emotional response and then returned to a neutral position. The duration of the emotional response was noted in seconds and values were summed to establish the length of time participants spent with a non-neutral expression. Valence (positive/negative) and the level of intensity were scored on a Likert scale (1–5; 0 was inputted if no positive/negative emotional expression was present). This was repeated for every emotional expression, providing overall totals for the frequency and duration of positive and negative emotional expressions and their mean intensity.

Coders also rated the level of interest, sadness, anger, happiness, fear, amusement and disgust observed during the ride on a Likert scale (1–6). Finally, coders selected the predominant emotion expressed throughout the ride from a choice of interest, sadness, anger, happiness, surprise, fear/disgust and neutral/indifferent.

Additional coding was included to evaluate features characteristic of CWA. Coders noted the frequency with which a participant pointed at something of interest and greeted or waved at other people, and as the aim was to capture the natural experience, the number of times the participant looked at the camera. Finally, how talkative participants were and how much discomfort they displayed during the ride was rated on a Likert scale (1–4).

### Statistical Analyses

As there were two independent coders, the intraclass correlation coefficient was used to measure reliability for each item. Mann-Whitney U tests were conducted to assess gender differences in the frequency, duration and intensity of positive and negative emotional expressions and Wilcoxon Signed Rank tests for differences in the frequency, duration and intensity across positive/negative emotional expressions.

## Results

### Rater Agreement

Intraclass correlation coefficients (ICC) computed across both raters for the FACES variables of frequency, duration, intensity, and levels of interest, sadness, happiness, anger, fear, amusement and disgust were high (mean ICC = 0.88, CI = 0.73–0.97). ICC were also computed for the number of times a participant pointed, greeted/waved and looked at the camera, and level of talkativeness and discomfort; rater agreement was again high (mean ICC = 0.83, CI = 0.51–0.95). Ratings were therefore averaged across raters for analyses.

### Facial Expression Coding

FACES ratings are shown in Table 1; in general, women displayed more emotional expressions (both positive and negative), and experienced them for longer and more intensely, though none of those gender differences were significant.

**Table 1.** Demographics and Emotional Ratings.

					Gender comparisons	
		Total sample (*n* = 12)	Females (*n* = 6)	Males (*n* = 6)	Mann-Whitney U	*p*
Demographics	Age	81.8 (7.4)	84.0 (9.4)	79.5 (4.5)	27.0	.180
	Gender (% Female)	50%	**—**	**—**		
	No. of previous CWA rides	8.0 (13.6)	5.2 (3.7)	10.8 (19.4)	21.0	.699
FACES ratings	No. positive	23.7 (13.5)	24.3 (7.5)	23.1 (18.5)	21.5	.589
	No. negative	0.4 (0.7)	0.6 (0.9)	0.2 (0.3)	20.0	.818
	Intensity positive	1.9 (0.4)	2.0 (0.4)	1.8 (0.4)	21.0	.699
	Intensity negative	0.3 (0.5)	0.4 (0.6)	0.3 (0.4)	20.0	.818
	Duration positive	139.6 (101.3)	140.4 (67.7)	138.8 (134.2)	20.0	.818
	Duration negative	1.3 (2.2)	1.7 (2.9)	0.9 (1.5)	19.0	1.000
	Interest	4.6 (1.6)	4.8 (1.7)	4.4 (1.7)	22.5	.485
	Happiness	4.7 (1.4)	5.3 (0.8)	4.2 (1.6)	27.0	.180
	Amusement	4.1 (1.5)	4.3 (1.3)	3.9 (1.9)	18.5	1.000
	Sadness	**—**				
	Anger	**—**				
	Fear	1.1 (0.4)	1.3 (0.6)	1.0 (0.0)	15.00	.699
	Disgust	**—**				
Additional ratings	Point	10.0 (7.6)	13.3 (8.1)	6.8 (6.1)	27.0	.180
	Greet	5.1 (3.5)	7.0 (2.8)	3.2 (3.1)	**31.0**	**.041**
	Look at camera	3.2 (3.2)	2.5 (1.5)	3.8 (4.3)	17.0	.937
	Talkative	3.5 (0.7)	3.6 (0.4)	3.4 (1.0)	15.5	.699
	Discomfort	1.3 (0.5)	1.4 (0.7)	1.2 (0.4)	21.5	.589

Note. FACES = Facial Expression Coding System. No. positive/negative = frequency of respective emotional expressions observed during the ride; Intensity positive/negative = mean intensity of respective emotional expressions, each expression having been rated on a scale of 1-5 with 0 inputted where none were present; Duration positive/negative = total time in seconds of respective emotional expressions observed during the ride. Interest/Happiness/Amusement/Sadness/Anger/Fear = level of respective emotion observed during the ride rated on a scale of 1-6; no instances of sadness, anger or disgust were recorded (M=1.0, SD=0.0). Point/Greet/Look at camera = frequency of respective behaviour observed during the ride; Talkative/Discomfort = level of respective behaviour observed during the ride rated on a scale of 1-4. Gender differences were analysed with Mann-Whitney U tests.

The number of positive emotional expressions ranged from 3–58 with a mean of 23.7 (SD = 13.5), which was significantly greater (*Z* = −3.061, *n* = 12, *p* = .002) than the number of negative emotional expressions (range 0–2, M = 0.4, SD = 0.7). Similarly, the mean intensity of positive emotional expressions (M = 1.9, SD = 0.4) was significantly higher (*Z* = −3.059, *n* = 12, *p* = .002) than negative emotional expression intensity (M = 0.3, SD = 0.5). In terms of duration, positive emotions were observed for a mean of 139.6 seconds (SD = 101.3) and negative for a mean of 1.3 seconds (SD = 2.2); this difference was significant (*Z* = −3.059, *n* = 12, *p* = .002).

None of the participants expressed sadness, anger or disgust. The only negative emotion expressed was fear (M = 1.1, SD = 0.4), considered very low according to the scale and only observed in females. Happiness (M = 4.7, SD = 1.4) was the emotion expressed to the highest degree. Interest (M = 4.6, SD = 1.6) and amusement (M = 4.1, SD = 1.5) were also rated highly across the sample. Females were generally rated as displaying each emotion to a higher degree, though gender differences were not significant (see Table 1). In terms of the emotion that was predominantly expressed throughout the recordings, both happiness and interest were most frequently selected by the raters.

In relation to the additional ratings, participants pointed to something of interest an average of 10.0 times (SD = 7.6) during a ride. They greeted or waved at others an average of 5.1 times (SD = 3.5) and looked at the camera an average of 3.2 times (SD = 3.2). Participants were talkative throughout their rides (M = 3.5, SD = 0.7) and displayed little to no discomfort (M = 1.3, SD = 0.5). There were no gender differences in these experiences (see Table 1), except for greeting/waving at others: females greeted others significantly more often than males (M = 7.0 (2.8) versus 3.2 (3.1), *U* = 31.0, *p* = 0.041).

## Discussion

The current study used an innovative methodology that provided novel insights into the generally positive experience that older adults have when participating in a Cycling Without Age ride. The advantage of video recordings is the observation of the experience in real time, without being influenced by the presence of a researcher or recall bias in interviews or surveys. Although a camera was attached to the trishaw, participants only looked at this an average of 3 times over their 30–40-minute ride, and these brief glances predominantly occurred within the first few minutes after the camera had been attached. Though participants may have been somewhat conscious they were being filmed throughout, the approach appears feasible for this and similar activities, adding value to the other methods of data collection often employed (Gow et al., 2019; Gray & Gow, 2020; McNiel & Westphal, 2020; Salas, 2018).

Findings support a positive emotional experience during a CWA ride. This is consistent with previous research reporting benefits in terms of mood and wellbeing, though those were predominantly collected as perceptions of the experience via interviews conducted some time after a ride had been completed (Gow et al., 2019; McNiel & Westphal, 2020). The positive emotions experienced during a ride likely underpin those positive reflections, and equally, are consistent with quantitative research regarding CWA’s immediate impact on the mood and wellbeing of participating older adults (Gray & Gow, 2020). Gender differences were observed in which females waved and greeted others more, and displayed longer and more intense emotional expressions in both positive and negative valence, though the latter differences were not significant. Findings have suggested that males may experience heightened emotional responses, whereas females express those emotions more intensely (Deng et al., 2016). This may present a limitation in using expressiveness as a metric of experience, and future research would benefit from coupling ratings of expressiveness with self-rated perceptions of the experience. The current study was also about the feasibility of assessing emotional experiences while limiting researcher interference, so any additional triangulation of methodology would need to be considered carefully in terms of introducing other recollection biases, for example. The current approach highlights aspects within the CWA experience that may have a causal link with the positive changes reported elsewhere.

There were very few negative emotions experienced during CWA rides, with only fear and discomfort being observed. Given the study population, it was expected that there may be some reports of discomfort, particularly when cycling over uneven ground, speed bumps, or mounting and dismounting curbs. It is worth highlighting, however, that very few participants experienced any level of discomfort. Similarly, few participants expressed fear, and those who did were often newcomers to the initiative and their fear was observed briefly at the outset of their first ride. More commonly, those taking part in the rides tended to be engaged with their surroundings, pointing at things of interest and greeting local citizens (often these greetings resulted in lengthy conversations) as well as being talkative in terms of conversations with both the other passenger and pilot. These results point towards the shared experience of those involved and highlights the social aspect of the initiative, which has similarly been identified from previous qualitative research (Gow et al., 2019; McNiel & Westphal, 2020). CWA therefore provides participants the chance to make new friends during trishaw rides. The shared experience while engaging with the local community, which has been observed to increase feelings of social inclusion for older adults when public transport is made more accessible (He et al., 2020), may partly explain the positive impact on mood and wellbeing. Similarly, group interventions have been highlighted as beneficial for older adults in residential care (Haslam et al., 2010).

While assessing the feasibility of a novel method for capturing in-activity experiences, the study does have several limitations. As with other CWA research, the sample size was necessarily small given the additional support needs of the population being considered. This was somewhat due to constraints regarding frequency of CWA activities, and the willingness to be recorded. The sample size therefore precluded a consideration of whether factors such as age or previous experience of the activity may have moderated emotional reactions. Both these factors varied greatly across the sample, so larger sample sizes will be required to assess their impact. For example, future studies including participants with a range of previous experience will be able to consider whether greater exposure to the activity is associated with a continual benefit or if there is a plateau in terms of positive emotional reactions. Other moderators to be considered include, but are not limited to, symptoms of anxiety/depression, sociability and personality. Though the current sample size reflected the exploratory nature of the study, larger scale trials are being planned given the feasibility of recording during the activity.

The results, therefore, provide a framework that can be scaled-up regardless of location. Ratings were sometimes more difficult depending on the seating position of participants and camera angle, which could move during rides. However, inter-rater reliability was high, suggesting FACES is an effective tool. The ratings were brief, which though advantageous may not cover all potential emotional experiences. Future studies might consider triangulating methods within a single study and assessing participants over longer periods to determine whether emotional experiences change with greater exposure and how those experiences translate to both short and longer-term impacts on mood and wellbeing. Future research may also aim to analyse the conversations between CWA pilots and passengers.

This brief study highlighted the positive experiences that older adults living in residential care had when taking part in CWA. Engaging in a shared experience and meeting new people is at the core of CWA, and the use a video-based research design allowed researchers to witness this first-hand. Future research should capitalise on this methodology when analysing CWA and other dynamic activities with older adults.
